# Integrating living biomaterials into neuroelectronic systems

**DOI:** 10.1007/s13534-026-00557-0

**Published:** 2026-02-20

**Authors:** Minseong Hong, YeongSeok Ye, Joungwon Kim, Jae-Ick Kim, Jong-Cheol Rah, Youngbin Tchoe

**Affiliations:** 1 Department of Biomedical Engineering, Ulsan National Institute of Science and Technology (UNIST), Ulsan, Republic of Korea; 2 Department of Biological Sciences, Ulsan National Institute of Science and Technology (UNIST), Ulsan, Republic of Korea; 3 Sensory and Motor Systems Neuroscience Group, Korea Brain Research Institute, Daegu, Republic of Korea; 4 Department of Brain Sciences, Daegu Gyeongbuk Institute of Science and Technology (DGIST), Daegu, Republic of Korea

**Keywords:** Biohybrid, Neural Interfaces, Microelectrode array, Living biomaterials-integrated neuroelectronics

## Abstract

Neural interface technologies stand at the threshold of a revolution, offering new possibilities for seamless, high-bandwidth interconnection between the human brain and computers. Recent progress has been driven by advances in microscale manufacturing, yielding sophisticated neural probes with diverse form factors capable of recording from macroscopic networks down to single units. These platforms span rigid-to-soft architectures and combine inorganic and organic materials, improving compatibility with the brain’s mechanical and chemical properties. Despite these advances, the field still relies primarily on nonbiological electrodes, which face inherent limitations in adapting to the dynamic and complex nature of living neural tissue. Living biomaterials-integrated neuroelectronics, on the other hand, could open new possibilities by enabling technologies that adapt to the host environment, actively establish bidirectional interfaces, conform to living tissue, and support repair by leveraging the inherent regenerative and plastic capacities of living systems. This review provides an overview of recent progress, challenges, and emerging directions in the integration of living biomaterials with neuroelectronic systems. We frame biohybrid neural interfaces as the convergence of in vitro microelectrode arrays and in vivo brain interfaces and organize the review around three themes: (i) cell sources for device integration, (ii) advances in in vitro MEA platforms, and (iii) cell-integrated, living electrodes for in vivo neural interfacing. Considered jointly, the themes point to an integrated path to seamless, adaptive biohybrid neural interfaces.

## Introduction

Neuroelectronic interfaces are at the forefront of research aimed at bridging the electrophysiological signals of living neural systems and the digital signals of artificial devices, underpinning the innovation in brain-machine interfaces, neuroprosthetic control, and neuromodulation therapies [1–4]. Despite numerous substantial advancements made in this field, achieving stable, long-term integration with the nervous system remains a formidable challenge. In chronic applications, abiotic neural interfaces—composed of nonbiological materials and lacking morphological adaptability—encounter challenges to maintain stable, high-fidelity electrophysiological recordings as neuron density tends to decrease near electrodes [5, 6], and inflammatory microglia and astrocytes encapsulate electrodes [6–11]. Beyond these issues, the unpredictable placement of neuronal cell bodies relative to electrode sites fundamentally limits the fidelity of neural recording, as high-quality signal acquisition often depends on chance [12, 13]. Tissue micromotion against implanted probes, together with ongoing dynamic biological responses, further destabilizes the neuron–electrode interface, producing inconsistent signals that compromise the reliability of brain-machine interfaces and diminish the precision of neuroscientific studies [14].

In contrast, biohybrid neural interfaces that integrate living biomaterials—cells, spheroids and organoids— into neuroelectronics, offer a fundamentally transformative approach. By leveraging the intrinsic adaptability, regenerative capacity, and native signal-transduction of neurons, the biohybrid neural interfaces could yield interfaces that actively couple to the host brain’s neural networks [15–21], maintain stable and intimate contact with target neurons [15, 16, 18], dampen immune activation [16, 22–24], and even help the regeneration of host tissue [18]. Furthermore, by carefully selecting the cell type incorporated into the device, it is possible to establish cell type-specific synaptic connections that enhance the selectivity of neuromodulation [16, 18, 19]. Moreover, this approach could enable access to deep brain neuronal circuits via axonal projections and network relays, thereby avoiding the tissue trauma associated with implanting the penetrating depth probes [16, 19, 20, 24]. The spectrum of biohybrid neural interfaces extends from engineering/selecting living cells to integrating them with neuroelectronics in a controlled manner, guiding synaptic connections to targeted host neurons for recording and modulation. The biohybrid paradigm in the neural interface field is reshaping how we conceive the device–tissue interface.

A critical foundation for the evolution of biohybrid neural interfaces is its close connection to in vitro microelectrode array (MEA) technologies, as biointegration in biohybrid neural interfaces typically begins with in vitro cell seeding and culture on neural probes prior to implantation. Decades of research in micro- and nanoscale engineering, particularly through microelectromechanical systems (MEMS), have yielded in vitro MEA platforms with unprecedented capabilities, including both extracellular [25, 26] and intracellular [27–29] multichannel electrophysiology, ultra-high-density arrays for subcellular-level mapping [30], and three-dimensional flexible architectures [31–33]. These platforms have become essential testbeds, enabling precise investigation of neural network function and fundamental mechanisms of cell–device interaction under controlled conditions.

In parallel, the other crucial foundation for biohybrid neural interfaces heavily incorporates the advancements in in vivo brain interface electrodes. Brain interface technology has advanced for decades to capture reliable local-field potentials and single-unit spikes from host neurons [34]. Form factors have diversified from rigid penetrating microelectrodes [35] to flexible polymer depth probes [2, 10, 36], and from traditional surface electrodes such as electrocorticography (ECoG) grids to flexible—and even stretchable—surface microelectrode arrays that conform to the cortical mantle [37–40].

**Fig. 1:**
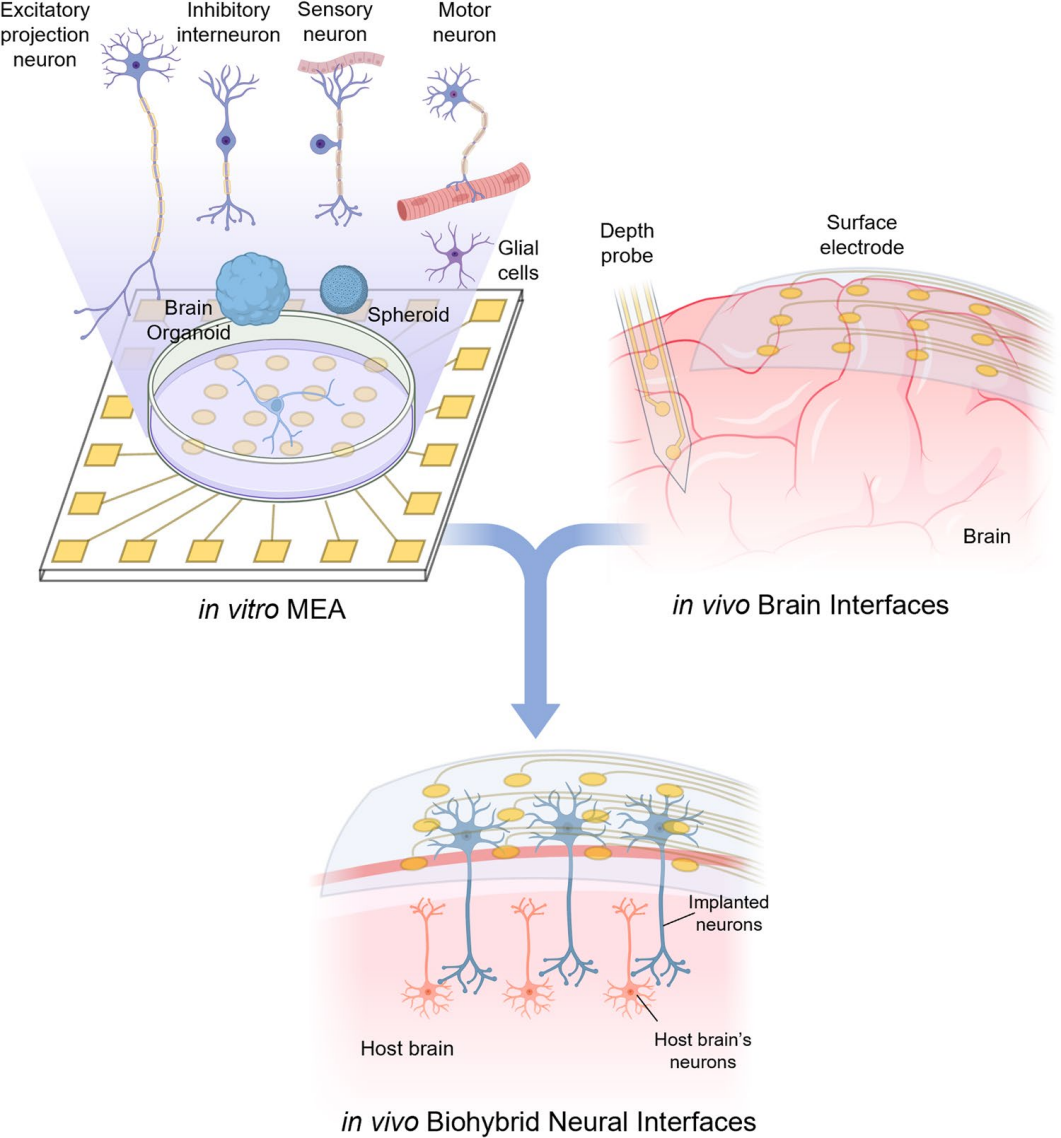
Overview of the biohybrid neural interfaces that combine innovations of in vitro MEAs and in vivo brain interfaces

The two paths now converge in biohybrid neural interfaces—combining in vitro cell culturing on MEA with in vivo implantable brain interfaces—creating living electrodes that merge biological components with electronics to deliver stable and adaptive interfaces (Fig. 1). Accordingly, this review is structured to connect MEA-derived capabilities with the cutting edge in vivo neural interface technologies, and to show how their convergence defines biohybrid system architectures.

To bridge the gap between in vitro MEAs and in vivo biohybrid neural interfaces, this review maps the landscape of living biomaterials in neuroelectronics from three linked vantage points. First, we survey neural cell sources—including engineered variants and structured assemblies—that underpin biohybrid strategies. Next, we assess state-of-the-art in vitro MEA platforms and consider how their architectures can inspire future biohybrid neural interfaces. Finally, we examine the rise of biohybrid neural interfaces for in vivo applications for seamless, durable, and adaptive interfaces. Building on prior reviews—linking cell transplantation with MEA technologies [41] and framing the field through biomimetic/bioactive/biohybrid approaches [42]—we present a technical and practical guide for engineers to review existing biohybrid neural interface system architectures. We would like to link the conceptual and technological continuum—from biological building blocks and bench-top MEA platforms to implantable biohybrid neural interfaces—and identify the key challenges and opportunities that chart a path toward neuroelectronic systems integrated with living biomaterials.

## Cell types for MEAs and biohybrid neural probes

The selection of appropriate cell types is a fundamental determinant in the design and performance of biohybrid neural interfaces, as different cell types enable functions that are unattainable with conventional nonbiological interfaces. It is important to consider cell types that integrate locally, project to defined targets, provide a clear excitatory or inhibitory output, and maintain layer- and target-specific connectivity. The chosen cell type should then be matched to the deployment format: excitatory projection neurons, inhibitory interneurons, sensory neurons, or motor neurons (with supportive glia) may be organized as planar 2D cultures for throughput and patterning; as spheroids that readily cast aligned axon bundles; or as organoids/assembloids that establish layered cytoarchitecture and form functional host–graft links. Source selection should consider synaptogenesis kinetics, cell supply, batch-to-batch reproducibility, cost, and time to experimentation. Early induced pluripotent stem cell (iPSC)-derived neurons or progenitors favor outgrowth and *de novo* integration but typically carry higher cost and longer lead times. Neural progenitor cells (NPCs) offer a balance between expandability and on-device differentiation at moderate cost and timelines. Primary embryonic neurons wire quickly with lower cost and faster deployment yet are limited by non-expandable, variable supply. Finally, programmability via optogenetic stimulation, Ca^2^⁺ imaging, and chemogenetic switches enables cell type-specific control with population-scale readouts. We survey the principal neuronal and glial types—and their engineered assemblies—used in biohybrid neural interfaces, outline practical sourcing routes, and highlight engineering strategies that enhance performance under multimodal recording and modulation.

### Types of neural cells

#### Excitatory projection neurons

Based on neurotransmitter type and anatomical connectivity, the major neuronal populations in the brain can be classified as either excitatory projection neurons, which primarily use glutamate or acetylcholine, or local inhibitory interneurons, which utilize γ-aminobutyric (GABA) or glycine. Excitatory projection neurons relay information across cortical layers and to distant targets [43], making them well suited for placement in biohybrid neural interfaces that bridge brain and electrode. Their expansive dendritic arbors sample convergent excitatory and inhibitory activity from local and long-range sources, while their long axons provide stable conduits to deliver signals to downstream targets. Long-range growth can, in principle, be steered by genetically encoded axon-guidance programs [44] and graded molecular cues, then refined by activity-dependent mechanisms [45] to form targeted, topographic projections. In cell-bearing biohybrid neural interfaces, the soma of a projection neuron can be anchored at an optical or electrical interface while its long axon is routed through microengineered conduits to transmit signals. This geometry helps preserve conduction under micromotion and mitigates mechanical mismatch and encapsulation, improving fidelity and longevity relative to conventional devices [16]. The configuration can also be reversed, with the soma on the host side to capture brain activity and route it to external hardware. Representative sources include primary rodent cortical pyramidal neurons for foundational studies [46] and human iPSC-derived cortical excitatory neurons for translational relevance [47].

#### Inhibitory interneurons

Unlike long-range projection neurons, inhibitory interneurons could fine-tune the signals locally. By forming localized contacts with defined subcellular compartments on adjacent cells, they regulate balance, timing, and signal filtering, thereby controlling which neuronal activity propagates and when [48, 49]. Their fast-spiking, perisomatic inhibition regulates oscillatory synchrony and temporal precision, maintaining the excitation–inhibition (E–I) balance essential for stable cortical computation and behavior [48, 50]. Because they can powerfully suppress and modulate specific circuits, inhibitory interneurons have been frequent targets for optogenetic manipulation in animal models [51]. Optogenetic activation of parvalbumin-positive interneurons provides a precise means of suppression, delivering fast perisomatic inhibition that reduces stimulus-evoked cortical firing and sharpens temporal precision without the broad spread typical of electrical silencing [51]. Although direct placement of inhibitory interneurons in biohybrid neural interfaces has not yet been reported, an excitatory biohybrid conduit targeted to an interneuron-rich layer preferentially formed synapses onto inhibitory cells, yielding robust inhibition with layer- and cell-type specificity beyond conventional electrical stimulation [52]. Therapeutically, engineered inhibitory circuits may be applied to compensate for E–I imbalance observed in neurological and psychiatric disorders such as epilepsy [53], schizophrenia [54], and autism spectrum disorders [55], offering a biologically grounded route for selective circuit modulation.

#### Sensory neurons

Neurons in the brain can also be classified according to their function as sensory neurons or motor neurons. Sensory neurons convert external stimuli—such as touch, light, temperature, or pain—into patterned spike trains, the native language of neural circuits [56, 57]. Because of this intrinsic specialization, sensory neurons are particularly valuable in biohybrid neural interfaces designed either for restoration of sensory pathways or for interrogation of sensory coding. With known stimulus–response spike trains [58, 59], artificial inputs applied through cortical-projecting sensory neurons can potentially be used to evoke sensory experience. Their peripheral afferents follow intrinsic guidance programs and molecular gradients [60], with activity-dependent refinement to yield modality-specific topographic maps (e.g., retinotopy [61], tonotopy [62], somatotopy [63]) that link external receptive fields to precise targets in the central nervous system [64]. Structurally, sensory neurons consist of a specialized peripheral receptor ending, a long axon, and synaptic terminals in the central nervous system. In application of biohybrid neural interfaces, this wiring logic can be leveraged to deliver layer-addressable signals that specifically could send stimulus type and intensity. Two of the most commonly used sensory neurons are dorsal root ganglion (DRG) neurons, which serve as long-axon encoders of somatosensation [65], and retinal ganglion cells (RGCs), whose spike trains carry feature-rich visual codes [57]; leveraging these physiologies brings biohybrid neural interfaces closer to replicating the brain’s native perceptual signals. Given their long-range projection capacity, DRG neurons have been used in tissue-engineered nerve grafts (TENGs), achieving ~ 5 cm axonal conduction while preserving pathway integrity in peripheral nerve repair [66]. RGC spheroids have also been integrated into biohybrid neural interfaces for deep brain stimulation, extending aligned axon bundles through microengineered conduits in an aim to transduce electrical input into propagating spikes and deliver layer-targeted outputs [20].

#### Motor neurons

Whereas sensory neurons route external inputs to the brain, motor neurons convert neural activity into muscle contraction via long peripheral axons and neuromuscular junctions (NMJs), making them well suited for biohybrid actuation and reliable control signals [67]. At the final efferent stage of the motor system, the firing rate and timing of motor neurons determine muscle force and contraction timing [68]. Accordingly, in biohybrid neural interfaces, patterned electrical or optogenetic input can evoke graded, precisely timed, and naturalistic muscle contraction to support motor recovery following injury. Recent studies using MEA platform have demonstrated that human iPSC-derived motor neurons can form functional neuromuscular junctions (NMJs) with engineered human muscle [69], highlighting the potential of biohybrid motor units to generate naturalistic outputs and provide reliable control when interfaced with electrodes for prosthetic or restorative applications.

#### Glial cells

Glial cells are non-neuronal cells that provide structural, metabolic, and protective support to neurons. They regulate immunity, homeostasis, synaptic function, and myelination, influencing the long-term integration and performance of biohybrid neural interfaces [70]. Although glial cell components are not yet incorporated as actively as neurons in biohybrid neural interfaces, their inclusion represents an important direction for future research and development. In current brain implants, the prevailing practice is to modulate host astrocytes and microglia—using compliant mechanics and surface chemistries—which helps moderate reactivity and preserve signals over weeks to months [71]. On the other hand, based on biohybrid approach, De Faveri et al. developed an electrode coated with the mixture of neurons and glial cells embedded in fibrin hydrogel to effectively hide the foreign object to the host tissue. They found that this coating does not alter the electrochemical properties of the electrode while showing markedly reduced tissue inflammatory reaction [23]. Not only in the brain but also in the peripheral nervous system, Schwann cell–seeded or Schwann-inductive conduits consistently improve axon regrowth and conduction across nerve gaps in vivo [72], indicating a clear path toward active glial incorporation. Overall, the evidence underscores the importance of biohybrid designs that integrate a robust mixture of neuronal and glial cells to promote stable integration and long-term viability in next-generation neural interfaces.

### Cell structure

#### Planar cell culture

Planar 2D cultures grow dissociated cells as a mono- or few-layer sheets on glass or plastic substrates, where the cells adhere to a flat surface and are maintained by media exchange from above. This format is simple, standardized, and inherently high-throughput [73]. For in vitro MEA studies, 2D cultures pair naturally with planar/CMOS arrays, supporting robust spike and field-potential readouts, closed-loop stimulation, and reproducible, statistically powered drug screening [74, 75]. As MEA densities and readout electronics improve, these platforms can capture fine-scale spatiotemporal patterns in dissociated neuronal networks while keeping workflows fast and scalable [75]. Owing to their simplicity and high throughput, planar 2D cultures remain the primary route for integrating living cells into biohybrid neural interfaces.

#### Spheroids

Neural spheroids are self-assembled 3D aggregates (typically hundreds of micrometers) that develop spontaneous network bursts and microcircuit architectures in a compact, diffusion-limited tissue mass [76]. They are employed when more physiological cell–cell interactions are desired than those offered by 2D cultures, while still maintaining advantages in throughput, uniformity, and short maturation times for neural-interface testing [77]. Moreover, because spheroids often extend long, bundled axons into surrounding matrices or toward nearby electrodes [20], they support robust device coupling and long-range signal routing in biohybrid neural interfaces. Recent 3D MEAs even actuate and wrap around spheroids to provide near-360° coverage and intact-tissue recordings, supporting pharmacological testing and propagation mapping without the need for tissue slicing—capabilities that planar MEAs struggle to achieve [32, 78].

#### Organoids

Brain organoids are self-organized 3D tissues derived from stem cells that recapitulate aspects of human brain development and cytoarchitecture beyond spheroids (e.g., layered zones and diversified cell types) [79]. They are chosen when the scientific question requires human-specific developmental programs, mesoscale connectivity, or complex oscillations. As brain organoids mature, they exhibit progressively richer electrophysiological activity and increasingly structured oscillations, which can be resolved using high-density CMOS-MEAs with tens of thousands of channels [80–82]. In a biohybrid organoid–host graft model, MEAs enable longitudinal monitoring of electrophysiological coupling between the graft and adjacent cortex, with functionally integrated organoids displaying sensory-evoked potentials and phase-locked oscillatory synchronization consistent with nearby host tissue [21].

#### Assembloids

Assembloids are self-organizing 3D preparations created by fusing region-specific brain organoids—or by combining organoids with other specialized cell types—to reconstitute multi-node neural circuits in vitro [83]. In neuroscience, they are used to model developmental programs and long-range connectivity with causal readouts, including interneuron migration in forebrain assembloids [84], thalamocortical synaptic function and plasticity [85], cortico-spinal–muscle motor pathways that generate contractions [86], and even four-part ascending somatosensory pathways linking peripheral input to the cortex [87]. Neural interface technologies have been actively applied to assembloids for multimodal interrogation to study the ongoing activities via 3D MEA, optogenetic stimulation, and calcium imaging [33, 85, 88].

### Functional engineering and cell manipulation modalities

#### Optogenetic engineering

Optogenetics couples genetic targeting with optical stimulation and readout to control specified neurons with millisecond precision [89]. Mechanistically, light-gated ion channels and pumps enable selective optical control of neuronal activity: blue-activated channelrhodopsins (ChR2) depolarize membranes to evoke action potentials, whereas yellow- or red-shifted halorhodopsins (NpHR) and archaeorhodopsins (Arch/ArchT) hyperpolarize neurons to induce silencing [90]. In neural interfaces, optogenetics decouples cell-class targeting from geometry, enabling artifact-free perturbations during recording and multiplexed activation or silencing of opposing populations [91]. Micro-LED-integrated in vivo depth probes [92, 93] and surface probes [94, 95] are actively being studied in this field together with the wide adoption of optogenetically engineered cells in biohybrid neural interfaces [19]. In parallel, optogenetically engineered cells are actively being adopted in the field of biohybrid neural interfaces through seeding cells on the cortical surface [24], site-selective integration with cranial window [19], and long-range axonal guidance through the hydrogel tube [16].

#### Calcium imaging

Calcium imaging has become a central methodology for mapping neural population dynamics with cellular specificity [96]. Using synthetic dyes or genetically encoded indicators (e.g., GCaMPs), optical readouts of intracellular calcium transients reveal spiking- and synaptic-related activity across multiple spatial scales—from somata to dendritic spines—with widefield, two-/three-photon, and head-mounted miniature microscopes [97–99]. These measurements enable the identification of ensembles, the interrogation of synaptic and network plasticity, and the longitudinal tracking of development, disease progression, and pharmacological perturbations [100, 101]. In the neural-interface arena, calcium imaging complements microelectrodes and optical/electrical stimulators by visualizing activation footprints, providing ground truth for spike sorting and decoder training, and offering biomarkers for adaptive, closed-loop control [38, 102–104]. It further underpins the evaluation of biohybrid constructs and organoids, and supports safety assessments around implants via chronic monitoring of neuronal excitability and glial calcium signaling [105]. In the biohybrid context, neurons expressing calcium indicators were seeded onto the cortex, and two-photon calcium imaging monitored graft activity and the emergence of host–graft connectivity [24]. Despite its utility, calcium imaging provides an indirect, temporally filtered signal and offers lower temporal resolution than electrophysiological recordings; in addition, optical scattering limits imaging depth and photobleaching/phototoxicity remain significant concerns [106].

#### Chemogenetics

Chemogenetics employs engineered receptors to make specific neuronal populations responsive to a matching, otherwise inert drug, enabling activity to be bidirectionally modulated on demand even without the need for external hardware or optical stimulation [107]. The most common tools are designer receptors exclusively activated by designer drugs (DREADDs): hM3Dq receptors increase neuronal firing, whereas hM4Di receptors suppress it [108, 109]. More recently developed ligands, such as deschloroclozapine (DCZ) and JHU compounds, provide more potent and selective control and can be tracked using positron emission tomography (PET) for long-term and deep-brain studies [110, 111]. MEA studies have shown that these perturbations produce clear shifts in firing, bursting, and synchrony in human iPSC-derived networks for scalable assays [112]. In nonhuman primates, inhibitory DREADDs (hM4Di) activated by on-demand DCZ rapidly quenched bicuculline-evoked epileptiform activities and limited their propagation, as verified with implanted subdural ECoG electrodes, thereby outlining a potential path to chemogenetic therapy [113]. As recent in vivo brain interfaces are increasingly integrated with on-probe microfluidics for targeted drug delivery [114], pairing these technologies with chemogenetically engineered cells could potentially enable selective, on-demand neuromodulation even without the need for electrical or optical modalities.

### Cell sources

#### iPSC-derived cells

iPSCs are adult somatic cells reprogrammed to a pluripotent state and subsequently directed—through precisely timed growth-factor and small-molecule cues—into defined neural lineages. This approach enables the generation of standardized batches of human cortical excitatory and inhibitory neurons, sensory and motor phenotypes, and astrocytes for supportive layers [115]. For implants to biohybrid neural interfaces, earlier developmental stages are often favored because their greater plasticity [116], neurite outgrowth, and synaptogenic capacity enhance survival and increase the likelihood of forming functional connections with the host brain [117]. In contrast, fully mature neurons tend to integrate less readily. iPSC-based approaches are particularly compelling for active incorporation into host neural networks and are advantageous when standardization, human relevance, or genetic tractability is prioritized, although they are typically more costly and time-consuming than NPC- or primary cell-based approaches. Beyond standardized human cell batches, patient-derived iPSC neurons and glia or other autologous sources suggest a path toward personalized biohybrid neural interfaces. In principle, patient-matched constructs could improve long-term compatibility by reducing immune mismatch and enabling tailored cellular composition and maturation state for host integration.

#### Neural progenitor cells

NPCs are expandable precursor cells from the central nervous system (CNS) that can be differentiated into neurons or astrocytes on demand, offering an intermediate option between fully pluripotent stem cell lines and fixed primary tissues [118]. For biohybrids, NPCs seed and differentiate well on hydrogel-based MEA microelectrodes and—especially when combined with electrical stimulation—promote nerve repair and functional recovery in a sciatic-nerve injury model [119]. NPC-based approaches are well suited for moderate-scale constructs that require faster timelines and on-device differentiation. They occupy a middle ground in both cost and time to experimentation between iPSC- and primary cell-based methods, and their expandable stocks shorten preparation time while reducing per-sample costs.

#### Primary neurons and glia

Primary neurons and glia can be harvested from embryonic or early postnatal rodents in a region-specific manner to match desired function, offering fast and physiologically relevant circuits for device testing, with clear trade-offs compared to iPSC/NPC models [120–122]. Embryonic forebrain tissue, particularly from the cortex, yields populations enriched in excitatory projection neurons suitable for long-range relay and stable decoding, while standard mixed cortical preparations also include inhibitory interneurons that contribute to local timing and synaptic filtering [123]. From the sensory periphery, DRG isolated from E15 or neonatal rodents provide primary sensory neurons [124]; from the spinal axis, embryonic spinal cord supplies motor neurons that extend long axons and form neuromuscular junctions [125]. In practice, primary cultures are rapid to prepare and relatively low in cost per plate; however, their fixed and variable supply, along with procurement scheduling constraints, limits scalability, motivating complementary use of iPSC or NPC sources for standardized, higher-throughput builds.

## Microelectrode arrays as in vitro platforms for biohybrid neuroelectronics

**Fig. 2:**
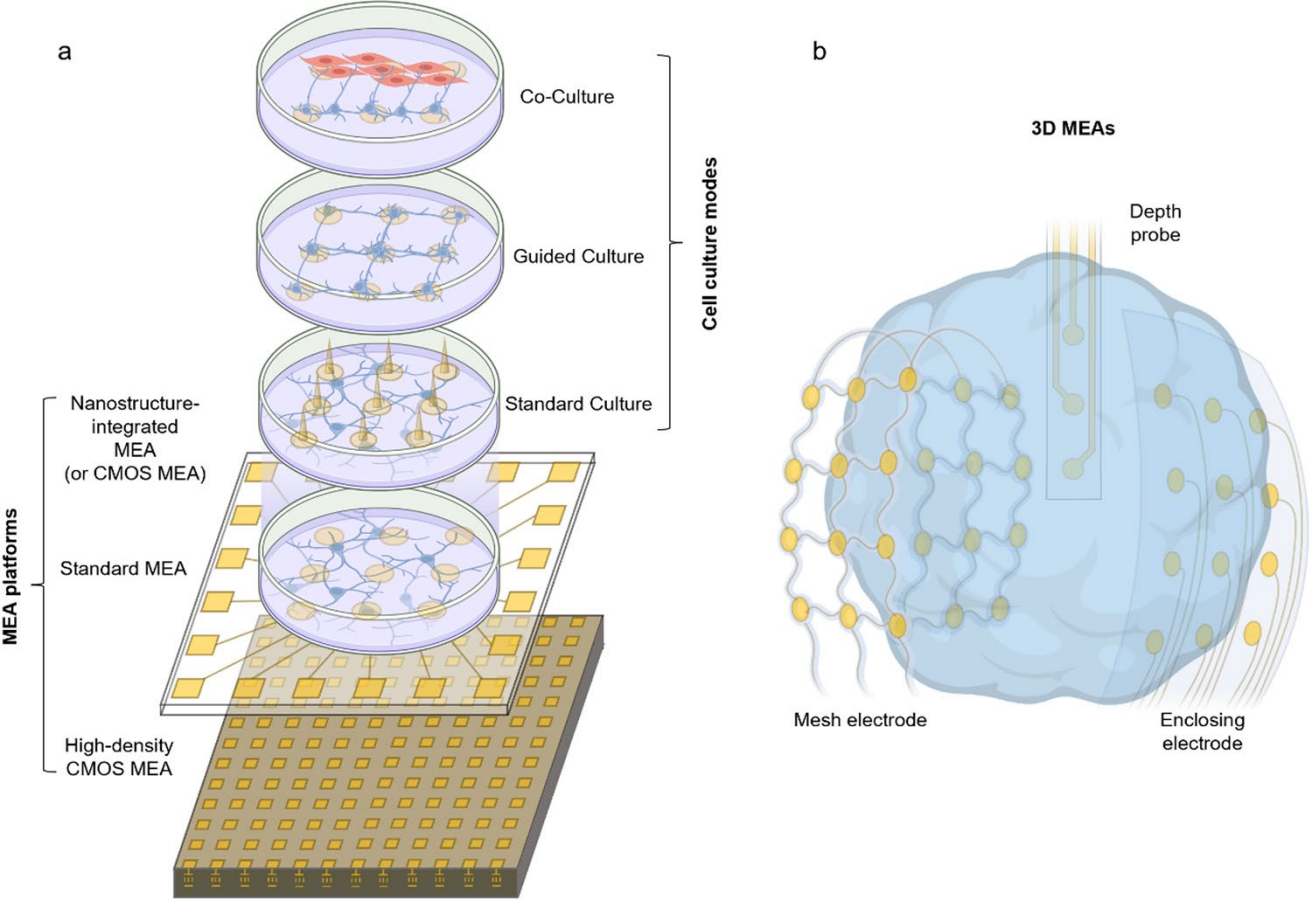
Schematic illustrations of system architectures of in vitro MEAs. **a** Various MEA platforms including standard planar MEA, high-density CMOS-MEA with fine pitch for subcellular-level spatial mapping, and nanostructure-integrated MEA for multichannel parallel intracellular recording. Different cell culture modes could be applied on these MEA platforms including standard culture, guided/patterned cultures constraining neurite growth and network layout, co-culture providing a testbed for functional connectivity between different cell types. **b** 3D MEAs such as mesh electrodes/depth probes/enclosing electrodes interfacing with cortical organoid

MEAs have transformed in vitro electrophysiology, moving beyond single- or few-cell patch-clamp assays to nondestructive, readily accessible network recordings, and have become an indispensable tool for drug discovery and basic research into neuronal communication [126]. Early photolithographically patterned planar MEAs allowed researchers to electrically monitor entire neural monolayers in parallel [25]. Advances in microfabrication have standardized device architectures, enabling reproducible multisite measurements across multiple wells [127]. This capability has made MEAs crucial for basic research, including the dissection of circuit dynamics [128], developmental processes [129], and synaptic plasticity [130], as well as for applied uses such as drug screening [131] and disease modeling [132]. We believe advances in MEAs have laid the groundwork for modern in vivo brain interface microelectrodes and are inspiring biohybrid neural interface systems that involve in vitro cell culture followed by in vivo implantation. Here, we review representative MEA modalities—planar arrays, guided and co-culture systems, nanostructure-integrated arrays, CMOS-MEAs, and 3D MEAs—highlighting techniques and findings that inform the design of future biohybrid neural interfaces (Fig. 2, Table 1).

### Guided neural circuits on MEAs

**Fig. 3:**
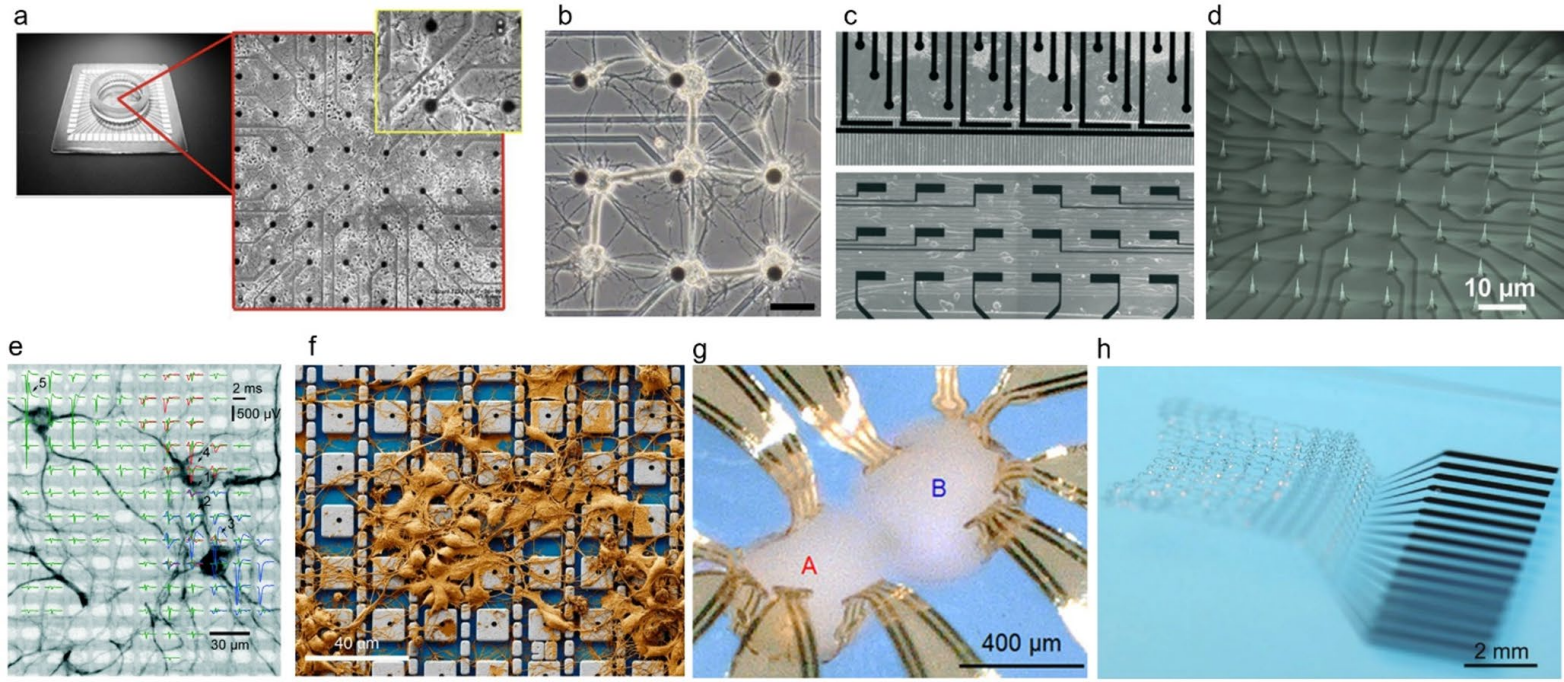
Various types of MEA platforms. **a** Planar MEA with rat cortical neuron culture [130]. Edited and reprinted with permission from Cadotte, A. J., et al., *PLoS ONE*, *3*(10), e3355, (2008). Copyright 2008 PLoS. **b** Guided MEA cultures in which cell clusters are patterned on individual electrodes, with neurite interconnections established via defined, dissolvable alginate hydrogel channels [133]. Edited and reprinted with permission from Lee, H., et al. *Biomedical Engineering Letters*, *13*, 659–670, (2023). Copyright 2023 Korean Society of Medical and Biological Engineering. **c** Co-cultured motor neurons (upper circle-shaped MEA) and myoblasts (bottom rectangle-shaped MEA) [69]. Edited and reprinted with permission from Duc, P., et al. *Lab on a Chip*, *21*, 4223–4236, (2021). Copyright 2021 Royal Society of Chemistry. **d** Vertically aligned ultrasharp nanowire array with sub 10 nm diameter for intracellular recording [134]. Edited and reprinted with permission from Liu, R., et al. *Advanced Functional Materials*, *32*, 2,108,378, (2021). Copyright 2021 Wiley-VCH GmbH. **e** An array of high-density CMOS-MEA measuring spikes from subcellular structures [30]. Edited and reprinted with permission from Müller, J., et al. Lab on a Chip, 15, 2713–2908, (2015). Copyright 2015 Royal Society of Chemistry. **f** Nanostructure-integrated CMOS-MEA for high-density, massively parallel intracellular recording [135]. Edited and reprinted with permission from Abbott, J. A., et al. Nature Biomedical Engineering, 4, 232–241, (2019). Copyright 2019 Springer Nature. **g** 3D MEA structure encasing and monitoring an assembloid [33]. Edited and reprinted with permission from Park, Y., et al. Science Advances, 7(eabf9153), (2021). Copyright 2021 American Association for the Advancement of Science. **h** Stretchable mesh electrode array that could incorporate within the cortical organoids [136]. Edited and reprinted with permission from Le Floch, P., et al. *Advanced Materials*, *34*, 2,106,829, (2022). Copyright 2022 Wiley-VCH GmbH

To extend the utility of MEA platforms, neural cultures can be artificially guided and patterned to form modular networks and enable functional connectivity (Fig. 2a). Guided neural circuits on MEA platforms use surface chemistry, microstructures, and fluidic compartmentalization to define network layout, direct axon growth, and impose region-specific connectivity in vitro [137]. Polylysines [138] or self-assembled monolayers [139] micropatterns confine somata and neurite trajectories over electrodes, enabling controlled layout and stable long-term recordings. These approaches support network-level phenotyping of spiking, bursts, and functional connectivity on standard and high-density MEAs. Microfluidic channels integrated with MEA devices can couple two compartments in a controlled manner via PDMS microtunnels and, with sequential plating to bias directionality, establish predominantly feedforward axonal links [140]. Furthermore, unlike permanent microfluidic channels, hydrogel-based dissolvable-barrier patterning extends this control: removable, cell-repellent alginate masks form electrode-aligned neuronal clusters that can be fused on demand by dissolving the barrier, yielding modular networks with enriched short-range functional connectivity, slower inter-cluster propagation, and more diverse network-burst motifs during maturation relative to random cultures (Fig. 3b) [133].

### Co-cultured cells on MEAs

Guided-culture MEA platforms could also make it possible to build structured co-cultures by combining complementary cell types. Duc et al*.* developed a human NMJ co-culture that integrates microfluidics, guidance microgrooves, and a custom MEA placing axon-stimulation and muscle-recording electrodes on the same chip, enabling presynaptic stimulation with postsynaptic readout (Fig. 3c) [69]. Patterning steered motor axons into aligned myotubes, producing robust NMJs verified by imaging and simple pharmacology. Axon stimulation reliably increased muscle spike counts and revealed distinct response patterns, demonstrating targeted motor-axon stimulation with simultaneous extracellular muscle recordings on-chip.

### Nanostructure integrated MEAs for intracellular recording

Despite the advantages of parallel multichannel recording capability of MEAs, the patch clamp-based intracellular recording is still the standard of electrophysiology. Nanostructure-integrated MEAs are progressively narrowing this gap by enabling parallel intracellular access while preserving multichannels electrical readout and compatibility with living networks [141]. Early work with vertical nanowire electrode arrays showed that membranes could be penetrated or tightly coupled to achieve single-cell resolution recordings and stimulation while remaining compatible with silicon fabrication and optical interrogation [142]. In parallel, engulfment-based gold mushroom microelectrodes (gMµEs) that is carefully designed to resemble the dendritic spine established in-cell–like recording without penetrating tips [29].

Advances in individually addressable vertical nanowire arrays further enhanced fidelity and stability. Nanowire-on-lead architectures aligned single nanowires to submicrometer interconnects, enabling intracellular spikes up to ~ 99 mV and pharmacologically verified postsynaptic potentials [28]. Further refiend ultra-sharp nanowires (sub-10 nm tips, ~ 7 µm height) allow sustained native intracellular recordings in neuronal and cardiac networks without electroporation, capturing graded pre-spike depolarizations and mapping tissue-level propagation (Fig. 3d) [134].

### CMOS-based high-density MEAs

The transition toward subcellular-level analysis in neural interfacing has been catalyzed by the advent of CMOS-based microelectrode array (CMOS-MEA) platforms. Built with advanced semiconductor foundry processes, CMOS-MEAs pack thousands to tens of thousands of individually addressable microelectrodes with local amplifiers onto a single silicon chip [26, 143]. The on-chip signal processing and multiplexing of CMOS-MEAs dramatically reduce electrical noise and data loss, supporting long-term, chronic recordings while allowing user-defined spatiotemporal control over stimulation and readout [30]. An electrode pitch as small as 17.5 μm enabled high-resolution mapping of electrical activity across specific axons, dendrites, and even subcellular domains (Fig. 3e). This unprecedented spatial and temporal resolution makes it possible to record fast action potentials, miniature synaptic events, and microdomain-specific activity simultaneously from hundreds to thousands of neurons. As a result, researchers can now resolve the initiation, propagation, and integration of signals through entire neural networks with massively parallel throughput [30]. Recent advances have extended CMOS-MEA applicability from monolayer cultures to patterned neural networks [144], human brain organoid slices [82], acute brain slices [143], and cortical organoids [81]. The CMOS-MEAs’ scalability, reliability, and resolution are setting new standards for functional connectomics and the direct investigation of subcellular computation within living neural circuits.

Nanostructure-integrated CMOS-MEAs extend conventional extracellular recording by providing intracellular-like access at scale, resolving subthreshold dynamics across thousands of interconnected neurons (Fig. 3f) [135, 145]. The Pt‑black coated 4096-pixel array integrates on‑site low‑noise amplifiers switchable between pseudocurrent‑clamp for membrane potential/PSP readout and pseudovoltage‑clamp for ion‑channel current assays, while continuous Faradaic current sustains gentle membrane permeabilization and stable access. The platform simultaneously recorded APs and PSPs from up to ~ 1800 pixels, mapped ~ 300 excitatory/inhibitory synaptic connections in 19 min, and quantified synaptic delays and propagation speeds, delivering patch‑like information with MEA‑scale throughput. Compared with traditional MEAs that miss subthreshold events, this CMOS-MEAs approach uniquely unites intracellular sensitivity, stimulation, and scalability for functional connectomics and high‑throughput pharmacology on neuronal networks. Further study has shown that CMOS-MEA integrated with platinum black microhole arrays could produce ~ 5 × larger action potential amplitudes and ~ 5 × longer coupling durations, while operating at ~ 5 × lower maintenance currents, than the nanoneedle arrays [146]. By minimizing electrode spread and nesting the microholes within wells, the design maximized seal resistance, enabling mapping of > 70,000 putative excitatory, inhibitory, mixed, and electrical connections across ~ 2000 neurons.

### Three-dimensional MEAs

Recent advances have transformed MEAs, shifting from flat arrays to three-dimensional (3D) architectures that conform to 3D cultures such as neural spheroids, brain organoids, and assembloids (Fig. 2b) [147, 148]. Key breakthroughs include ultraflexible polyimide-gold cage arrays that gently encapsulate organoids, offering minimally invasive integration and preserving tissue health through open meshwork designs that support nutrient flow and matrix elaboration (Fig. 3g) [33]. Configurations span single, dual, and multi-cage systems capable of interfacing with up to 16 fused spheroids, supporting assembloid fusion and long-term studies of neural maturation or disease modeling. Beyond electrodes surrounding the organoid surface, upright, depth-probe–like electrodes can record throughout the 3D volume within the cortical organoid; by actuating flexible polyimide probes to stand within the gel, researchers achieved stable recordings suitable for network mapping and pharmacological or disease studies [149]. Complementing these approaches, stretchable mesh conforms to organoid surfaces or integrates within the tissue, enabling minimally invasive, high-density recording and stable long-term monitoring across complex 3D architectures (Fig. 3h) [136]. These open, macroporous arrays can be delivered directly into soft neural cultures or tissues, promoting cellular ingrowth, reducing foreign body responses, and providing stable, long-term access for electrical recording and stimulation [136, 150].

**Table 1:** State of the art *in vitro* MEAs

Type	Core technology	Cell source/form	Channels	Impedance	Density (electrodes/mm^2^)	Key demonstration	References
Standard MEA	MEMS-based microfabrication	Primary and iPSC-derived cells	16–64	400 kΩ @ 1 kHz	2–200	Non-destructive, multisite extracellular monitoring, network analysis	[25] [46]
Guided culture MEA	Dissolvable hydrogel barrier guided culture	Primary rat hippocampal neuron	59	–	30	Dynamic control of connection between patterned clusters	[133]
Co-cultured MEA	Co-culturing of motor neurons and myoblasts	hiPSC-derived motor neurons & primary human myoblast-derived myotubes	59	–	6	Functional human neuromuscular junction formation in vitro	[69]
CMOS-based MEAs	High density IC with on-site amplification, impedance spectroscopy, cyclic voltammetry	Primary rat cortical neurons and mouse cerebellar slices	2048	800 kΩ @ 1 kHz	5487	Subcellular level spatial resolution	[143]
3D MEA	Flexible enclosing cage	hiPSC-derived cortical spheroids and assembloids	9–34	10 kΩ @ 1 kHz	≈28 electrodes/mm^3^	Surface signal monitoring	[33]
	3D upright MEA	hiPSC Glu/GABA neurons and astrocytes	256	40 kΩ @ 1 kHz	≈34 electrodes/mm^3^	3D volumetric signal monitoring	[149]
	Mesh electrode	hiPSC-derived cortical brain organoid	16	140 kΩ @ 1 kHz	≈14 electrodes/mm^3^	Recording within the cortical organoid	[136]
Nanostructure- integrated MEA	Gold mushroom resembling dendritic spine	Primary rat hippocampal neuron	60	–	100	Electroporation-enabled intracellular recording	[27]
	Ultrasharp Si nanowire array	Primary rat cortical neurons and iPSC-CVPCs	64–128	14.95 MΩ @ 1 kHz	200–4000	Natural internalization	[134]
	CMOS-MEA with PtB nanoneedles or microholes	Primary rat cortical neurons	4096	300 kΩ @ 5 kHz	2500	Electroporation-enabled parallel intracellular mapping	[135] [146]

## In vivo biohybrid neural interfaces

**Fig. 4:**
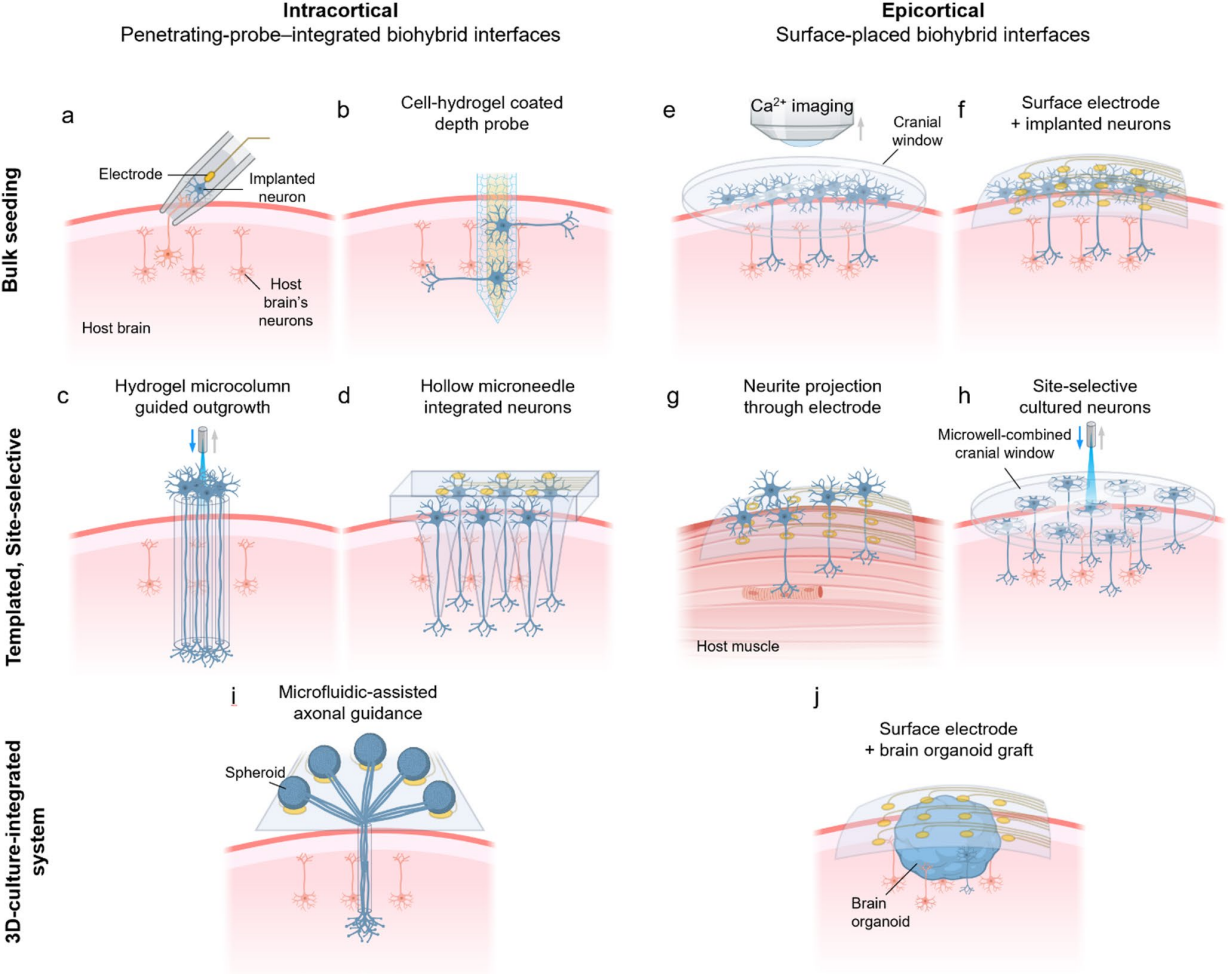
Schematic illustration of the system architecture and living-cell incorporation strategy of representative in vivo biohybrid neural interfaces. **a** A glass-cone electrode containing a sciatic-nerve graft implanted into the cortex, promoting host neurite ingrowth and interconnection with the graft. **b** Depth probe coated with hydrogel containing cells. **c** Hydrogel microcolumn with neurons at the inlet; axons are guided to the outlet, establishing a pathway that links the device to host tissue. **d** Microneedle array containing neurons where neurite outgrowth and connect with the host brain. **e** Cells seeded on the cortex and a transparent cranial window allowing calcium imaging. **f** Cells seeded on cortex and surface electrodes recording the electrophysiological activity. **g** Perforated surface electrode seeded with motor neurons where axonal outgrowth through the holes could form NMJs with the underlying host tissue. **h** Microwell-combined cranial window for site-selective culture of neurons for optical stimulation and readout. **i** Spheroid placed on electrode-containing wells on the cortex and axons guided from each surface well converging to a single point in the deep brain. **j** Brain organoid transplanted into the cortex and surface electrode interfacing with both the host and the graft

Conventional brain interfaces (abiotic neural probes), even in their most advanced flexible or soft forms, remain limited by the absence of active biological remodeling with host tissues. Biohybrid neural interfaces integrate living cells with electronics to promote adaptive coupling with host neurons and could participate actively within the host neural system. This section reviews state-of-the-art biohybrid neural interfaces with a primary focus on architecture and living-cell incorporation strategy. We organize the field along three design axes: (i) interface location—intracortical (penetrating-probe–integrated) vs. epicortical (surface-placed); (ii) cell deployment—bulk seeding vs. templated, site-selective seeding; and (iii) tissue format—2D overlays vs. 3D culture–integrated systems (Fig. 4, Table 2).

### Intracortical, penetrating-probe–integrated biohybrid interfaces

#### Glass-cone electrode with nerve integration

A key milestone in biohybrid neural interfaces was the cone electrode—a glass cone containing a gold microwire and a sciatic nerve, implanted into the cortex (Fig. 4a) [151]. Host brain’s neurites readily grew into the cone, forming an electrical interface with the embedded gold wire and demonstrated a stable recording for over months. The study reports long-term extracellular recordings spanning 11 months, with the characteristic observation that coupling can improve during the early post-implantation period as neurites populate the recording region and then remain comparatively stable over extended durations of up to ~ 1 year. It demonstrates a core principle of biohybrid neural interfaces where biological scaffolds guide neurite ingrowth, anchor neural processes, and prevent glial encapsulation to maintain signal quality for long-term. Whereas conventional electrodes often lose signals due to glial encapsulation and micromotion, the cone electrode leveraged endogenous integration for stable, long-term recordings. However, because incorporating the sciatic nerve into a glass cone electrode is challenging, more recent neurotrophic electrode studies have instead explored filling the glass cone with nerve growth factor to guide neurite ingrowth from the host [152, 153]. While limited to one channel, the cone electrode sets the paradigm that incorporating living tissue into the device can yield durable, natural brain–device coupling.

#### Depth-probe coated with neurons

**Fig. 5:**
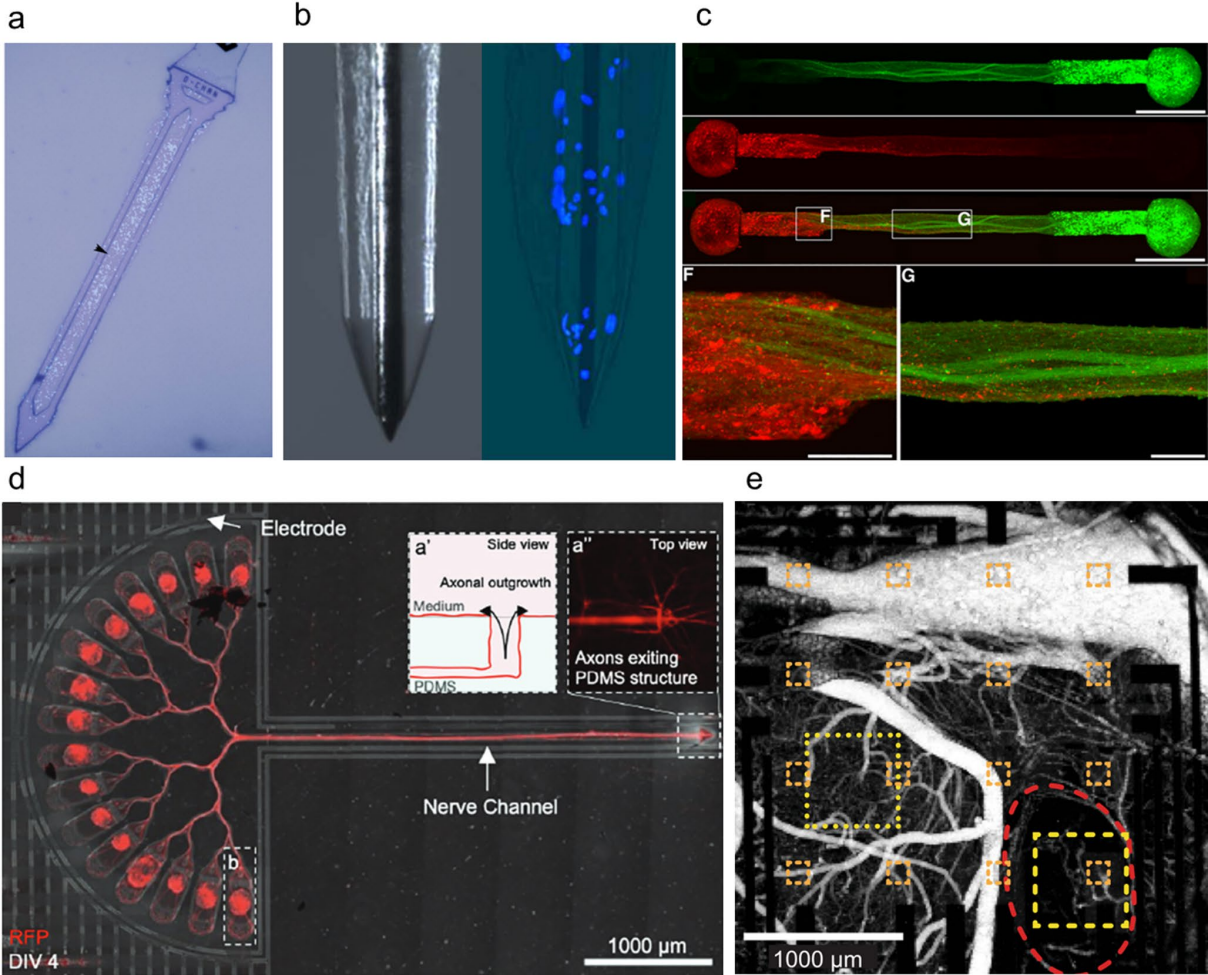
Various types of biohybrid neural interfaces. **a** A parylene-based device with a hollow well that contains NSCs within an alginate hydrogel [22]. Edited and reprinted with permission from Purcell, E. K., et al. Journal of Neural Engineering, 6, 026005, (2009). Copyright 2009 IOP Publishing. **b** Cell-hydrogel coated microwire with fluorescence microscopy image showing cells stained with DAPI [23]. Edited and reprinted with permission from De Faveri, S., et al. Frontiers in Neuroengineering, 7, 7, (2014). Copyright 2014 Frontiers. **c** Hydrogel microcolumn-based, micro-tissue engineered neural networks (µTENNs) where fluorescence microscopy images show cortical neuronal aggregates seeded individually at both ends. One end of the implant is fused with the brain, while the other end faces the optoelectronic light source or imaging sensor. (upper) monodirectional neurite projection from each end and (lower) bidirectional neurite projection joining together near the intersection [16]. Edited and reprinted with permission from Adewole, D. O., et al. *Science Advances*,* 7*(eaay5347), (2021). Copyright 2021 American Association for the Advancement of Science. **d** Spheroid-based biohybrid system for deep brain stimulation where individual spheroids are placed in 16 wells each with stimulation electrodes. Matrigel-coated PDMS guide facilitates guided axonal growth that extends toward the single point inside the deep brain region [20]. Edited and reprinted with permission from Sifringer, L., et al. *Advanced Functional Materials*, *35*, 2,416,557, (2025). Copyright 2025 Wiley-VCH GmbH. **e** Transplanted cortical organoid (circular red dotted line) with transparent electrode array (orange dotted square array). Vascular connections between the host and the transplant site can be observed [21]. Edited and reprinted with permission from Wilson, M. N., et al. *Nature Communications*, *13*, 7945, (2022). Copyright 2022 Springer Nature

Embedding living cells within penetrating probes aims to enhance biocompatibility and to stabilize long-term neural recordings by leveraging the cell’s vital activity and neurite outgrowth (Fig. 4b). Purcell et al. seeded neurosphere-derived neural stem cells (NSCs) at roughly 100 cells in the parylene probe well plus an additional approximately 500 cells in the exterior alginate hydrogel layer (Fig. 5a) [22]. Relative to controls, biohybrid approached samples at day 1 and week 1 showed higher neuronal density and reduced non-neuronal/glial presence near the implant, suggesting attenuated early glial encapsulation and a transient improvement in the peri-electrode cellular milieu. Importantly, these benefits were reversed after 6 weeks, with reduced neuronal density and increased glial encapsulation around NSC-seeded probes, which the authors attributed to declining NSC viability and inflammatory responses associated with alginate scaffold degradation and accumulation of cellular debris. Collectively, this work underscores that cell-based integration can introduce time-dependent biological trajectories—initially favorable but potentially diminishing or even adverse over weeks to months—rather than guaranteeing sustained improvements in chronic outcomes. De Faveri et al. further suggested advanced cell integration technology by developing fibrin-based cellular coatings (Fig. 5b) [23]. Their system employed primary hippocampal neurons and astrocytes embedded within controlled-thickness fibrin hydrogels. Four dipping cycles achieved optimal coating thickness that preserved electrode function while providing cellular benefits. In acute in vivo recordings, the fibrin-coated electrodes maintained spike recording quality comparable to uncoated controls (85% of units with SNR > 3), indicating that the added coating did not markedly compromise electrophysiological readout. Fibrin-coated electrodes showed reduced astrocyte reactions at both 7 and 30 days post-implantation, with progressive hydrogel reabsorption occurring within 7 days without problematic swelling. These results highlight a practical design principle for biohybrid neural interfaces, suggesting that permissive and rapidly resorbable cell-laden ECM-like coatings can modulate the early peri-electrode response while preserving electrical function.

The direct seeding of NPCs on silicon-based multichannel neural probes represents another important milestone in biohybrid neural interfaces [15]. Azemi et al. suggested to address a core obstacle in chronic neural recording—neuron loss near implants and reactive gliosis—by seeding silicon probes with adult rat NPCs on covalently laminin-immobilized surfaces to improve the tissue interface. In vivo, NPCs remained on and around the probe after a week of implant, and qualitative histology suggested a reduced GFAP astrocytic response versus controls. Overall, this work shows proof of concept that NPC-seeded, laminin-modified multichannel depth probes can improve acute biocompatibility and may stabilize the electrode–tissue interface, motivating long-term evaluations of chronic recording performance.

#### Hydrogel-tube-assisted guided axonal routing

In micro-tissue engineered neural networks (μTENNs), neuronal clusters at one or both tube ends could send aligned axons through a soft hydrogel core, forming a “living cable” that carries signals from deep brain sites to surface devices without rigid intracortical probes (Figs. 4c and 5c) [16]. By keeping the non-biological electronics outside the cortical surface, directing axonal outgrowth toward depth, and leveraging synapses formed at depth, these constructs act as biological relays that aim to both reduce chronic immune responses while enhancing signal selectivity through anatomically constrained, phenotype-tuned connections. In long-term culture, the viability of μTENNs was 80–85% at 28 DIV, which was higher than that of planar culture. Functionally, they expressed ChR2 for photostimulation at one end and RCaMP/GCaMP for calcium imaging at the contralateral end, and demonstrated that photostimulation induced time-locked calcium responses, verifying signal propagation/regulation along axonal tracks. After transplantation into the rat visual cortex, μTENNs demonstrated survival and spontaneous activity for several days to several weeks using multiphoton calcium imaging, and histological analysis at 1 week to 1 month showed relatively mild inflammatory reactivity along with host-oriented neurite outgrowth and putative synaptic integration. μTENNs introduce a structural separation between the biointerface and non-biological electronics. Yet, biomaterial-supported axonal bundles extend deep into the brain and form synaptic connections with host circuitry, minimizing immune responses and allowing surface-mounted electronics to access host circuitry. This approach goes beyond simple integration of biomaterials and electronics, addressing the biocompatibility, immune response and mechanical mismatch between the brain and electronics, a key issue in neural interfaces.

#### Microneedle guided axonal routing

A hollow, neuron-seeded microneedle platform has also been demonstrated as a guided biohybrid neural interface (Fig. 4d). Ahmed et al. reported a biohybrid transition microelectrode array (TMEA) that couples a 4 × 4 array of polymer microneedles to a silicon base bearing gold-coated μWells pre-seeded with prenatal murine cortical neurons [17]. Each microneedle contains an ~ 20 μm internal lumen filled with GelMA (gelatin methacrylate), intended to provide a permissive, guided path for neurite extension from the on-chip cell reservoirs into tissue. Conceptually, the neurites projecting through hydrogel-filled channels would synaptically couple to host circuits, forming a “living conduit” that links the electronics at the surface to deeper neural networks without relying on a rigid metallic tip at the target site. The base electrode part excluding the cell and hydrogel was evaluated by accelerated aging (67 °C PBS, 46 days ≈ 37 °C 1 year equivalent), and the impedance was maintained without significant change from ~ 16.5 to ~ 15.5 kΩ under 1 kHz. As an initial proof-of-concept, the authors showed neurite projection along the shanks and axon outgrowth into a hydrogel phantom, and separately validated the recording chain by capturing artificial 2 kHz spike-train inputs using unloaded devices. Although further verification is required because in vivo experiments have not yet been conducted, this study is noteworthy in that it achieved physical separation of biomaterials and electronics while maintaining the electrical properties of multiple channels [17].

### Epicortical, surface-placed biohybrid interfaces

#### Seeding cells on cortical surface

Seeding cells on cortical surfaces represents a promising biohybrid approach for accessing deep brain circuits without the trauma of penetrating probes (Fig. 4e and f) [24]. Rather than relying on stiff penetrating shanks, engineered neuron–glia constructs send neurites downward to form functional synapses with host neurons across layers, creating relay connections. Cultured neurons in this overlayer are interfaced both by Ca^2+^ imaging and by surface microelectrode recordings at the device. Critically, cell-seeded cortical interfaces can access deep brain layers via axonal relays from the surface avoiding insertion-related trauma, micromotion shear, and chronic gliosis that limit the longevity of penetrating microelectrode arrays [24]. A remaining challenge is that uncontrolled neurite outgrowth can yield nonspecific connections and diffuse signals. However, site-selective seeding and guided axonal routing may mitigate these issues.

#### Cell-seeded surface electrode

Beyond the strategy of seeding neurons onto cortex, Stieglitz et al. proposed a perforated microelectrode array placed on the cortical surface with a cell reservoir above it (Fig. 4g) [154]. In this architecture, each 40-µm perforation encircled by a ring electrode acts as a guided axonal growth conduit that could also both record and stimulate the neurites passing through the hole. As proof of concept, they demonstrated flexible two-layer polyimide electrode array carrying 19 iridium ring sites with perforations. Average impedance of each ring site is 15.5 kΩ at 1 kHz. This platform could be used for biohybrid grafts in which neuronal axons are routed through the perforations for targeted regeneration of neuromuscular or cortical connectivity.

Skeletal myocytes—rather than neurons—could be seeded onto biohybrid neural interfaces to promote regeneration of injured NMJs. Rochford et al. developed a flexible 32-channel parylene-C microelectrode array with PEDOT:PSS electrodes, coated it with a fibrin hydrogel seeded with human iPSC-derived myocytes, and cultured the construct until the cells matured into multinucleated myotubes [18]. The device also incorporated circular openings to facilitate fluid/cell exchange and vascular ingrowth, aiming to sustain graft viability after implantation. Over 4 weeks in a rat forearm nerve injury model, electrode impedance increased and functional yield declined (pre-seeding: 97% yield, 1.84 kΩ; week 4: 25% yield, 159.0 kΩ), yet the authors reported these changes to be acceptable and within expectation. In immunodeficient rats, histology confirmed survival of the transplanted hiPSC-derived myotube layer through 28 days with close apposition to the device. Notably, regenerated nerves were frequently observed near the biohybrid implant and robust NMJ markers emerged only in biohybrid groups, consistent with host motor axon innervation of the grafted myotubes. Functionally, at 4 weeks under anesthesia, 100-µA stimulation evoked no compound action potentials (CAPs) in control groups, whereas clear CAPs were detected with biohybrid groups, supporting improved coupling between regenerating axons and recording sites. In longitudinal awake recordings, signals were minimal during weeks 1–2 but rose sharply by weeks 3–4, with mean SNR increasing from 12.9 and 11.3 dB (weeks 1–2) to 19.7 and 32.0 dB (weeks 3–4). By connecting host and seeded cells with appropriate cell types, biohybrid neural interfaces can achieve higher SNR and high-quality signal recording. Furthermore, this study suggests these interfaces improve biocompatibility and can even lead to the regeneration of damaged host tissue [18].

#### Site selective cell seeding

Although bulk seeding of cells can yield highly viable grafts and increase the likelihood of host integration by sheer cell number, site-selective culture could enable precise intent-driven architecture and reproducible interface (Fig. 4h). Brown et al. presents a cortical-surface biohybrid implant that combines a cranial window-like optical interface with a high-density SU-8 microwell scaffold designed to hold one neuron per well, aiming to demonstrate a functional proof-of-concept for brain interfacing without penetrating probes [19]. The scaffold was fabricated with photolithography and bonded to a glass coverslip to maintain optically access for stimulation/imaging through the coverslip. By utilizing dissociated E14.5–E15.5 primary cortical neurons, the study leveraged the syngeneic source to frame an autologous graft concept that avoids immunomodulatory therapy in this mouse-stage implementation. They also note that the embryonic cortical source should be predominantly glutamatergic excitatory, and report no teratomas/overgrowth. DAPI-based occupancy at 24 h post-loading was 77 ± 15%, corresponding to 90,000 microwells containing neuron. After craniotomy/duratomy, the device was placed onto mouse cortex, and two-photon imaging showed no obvious structural deterioration of the scaffold even months post-implantation, supporting mechanical stability of the platform. After 3 weeks, in vivo two-photon imaging found 52 ± 23% of microwells contained neurons, interpreted as robust survival relative to typical CNS graft survival, and grafted neurons extended extensive processes into superficial cortex. Histologically, explanted arrays retained cells in microwells and showed vascularized tissue consistent with tight coupling, and limited sections supported robust superficial integration with axons observed across cortical layers. Critically, mice implanted with optogenetically transduced neurons learned to report stimulation of the graft in a behavioral task. Five of nine animals achieved criterion performance, demonstrating that cortical surface biohybrid neural interface with selectively seeded cells can provide input to guide goal-directed behaviour [19].

### 3D culture integrated platform

#### Spheroid-based targeted deep brain modulation

Compared with 2D cultures, neural spheroids offer a self-organized 3D microenvironment, robust axon bundling, and better surgical handling, making them attractive building blocks for biohybrid probes. Sifringer et al. demonstrated a biohybrid interface that couples neural spheroids to a stretchable microelectronic platform with guidance microchannels, relaying surface-evoked activity to deep targets while permitting axonal propagation (Figs. 4i and 5d) [20]. Each spheroid is placed in a microwell on an electrode, and passes through an axon-only barrier to enter a guidance microchannel. Axons from multiple channels converge into a single bundle that extends deep into the microchannel, serving as a living relay between spatially separated input (electrode stimulation) and output (target synaptic activity). This structure was maintained in vitro for 167 days. Functionally, selective electrical stimulation of spheroids could allow signal propagation along a pathway, with minimal leakage between adjacent wells. This electrode system exhibited an impedance of 8 kΩ at 1 kHz, which remained below 100 kΩ at 1 kHz even after accelerated aging and implantation. The authors reported that this impedance range is sufficiently low for neural activity recording. In vivo, spheroid survival and spontaneous activity persisted for weeks, and axonal outgrowth toward the target was observed within 3–5 days, indicating the feasibility of the approach. Despite these advances, axonal integration into host parenchyma and synapse formation has not yet been demonstrated, and axons degenerated after ~ 5 days in vivo, with glial infiltration suggesting immune rejection of xenogeneic spheroids. These outcomes underscore the need for immune management, and improved nutrient access—possibly via subdural placement or vascularized scaffolds—to achieve durable host coupling. Nevertheless, the results validate critical aspects: surgical feasibility, short-term axon extension into guidance conduits, and chronic spheroid survival.

#### Organoid-integrated biohybrid neural interface

Moving beyond spheroids, human brain organoids add layered, regionally patterned tissue with diverse cell types and developmental programs, making them compelling building blocks for biohybrid neural probes (Fig. 4j). Recent work has demonstrated that transplanted cortical organoids can vascularize within host cortex, extend axonal projections, and establish synaptic connectivity, raising the possibility of using organoids as implantable biological relays or prosthetic modules [155]. A notable advance came from Wilson et al., demonstrating the integration of human cortical organoids with transparent graphene microelectrode arrays for chronic in vivo monitoring [21]. In terms of electrode performance, 1 kHz impedance was ~ 1.4 MΩ pre-implant and most channels maintained stable impedances throughout the chronic study. When organoids were implanted into the mouse retrosplenial cortex and interfaced with these arrays, multimodal monitoring via electrophysiology and two-photon imaging revealed progressive vascularization, stable survival, and sensory-driven activity (Fig. 5e) [21]. Signal quality was sufficient to resolve single-trial visually evoked LFPs across channels, and by ~ 3 weeks post-implantation organoid-overlying channels exhibited stimulus-evoked LFP responses comparable in amplitude to surrounding cortex, consistent with functional coupling to host visual pathways. For spiking-band activity, spontaneous MUA rates remained relatively stable (~ 2 Hz) across the 8–11 weeks recordings, and stimulus-locked MUA modulation was quantified via peak SNR values reported in the 2.36–5.21 range across channels. Histological analysis further demonstrated bi-directional synapse formation between the human organoid graft and the mouse brain, with human presynaptic puncta extending into host cortex and host inputs penetrating the graft. Importantly, organoids also exhibited distinct physiological signatures under anesthesia, suggesting local integration but incomplete recruitment by long-range modulatory circuits. Together, these studies position organoid-based biohybrid electronics as a frontier for living electrodes that adapt over time and interface at the circuit level in vivo. By enabling longitudinal, multimodal tracking of organoid maturation and host connectivity, such platforms may support next-generation neural prostheses and brain–computer interfaces, where engineered organoids could help restore function in injured brain regions.

**Table 2:** State of the art in vivo biohybrid neural interfaces

Class	Core technology	Modality	Channels/wells	Impedance	Cell source/type	Key demonstration	References
Intracortical							
Penetrating-probe–integrated biohybrid interfaces	Glass-cone electrode with nerve graft	Electrical recording	1 channel	50–300 kΩ @ 1 kHz	Sciatic nerve	Host’s cortical neurites ingrowth into cone containing sciatic nerve and electrode wire	[151]
	Cell-hydrogel containing probe	–	1 well	–	E14 murine cortical NSCs	Parylene depth probe with an open well seeded with neural stem cells encapsulated in an alginate hydrogel scaffold	[22]
	Cell-hydrogel covered probe	Electrical recording	1 channel	200 kΩ @ 1 kHz	Primary hippocampal neurons (E18 rat embryos) and glial cells (P0 rat pups)	Penetrating needle coated with resorbable fibrin hydrogels containing neurons and glial cells	[23]
	Hydrogel tube guided axonal growth	Optical stimulation & calcium imaging	–	–	Primary cortical neuron	Bi-directional axonal tracts projection within soft hydrogel cylinders. Optobiological monitoring/modulation of brain activity	[16]
	Microneedle-guided axonal growth	Electrical recording & stimulation	16 channels	15.5 kΩ @ 1 kHz	Primary cortical neuron	Neurons embedded on a silicon-based chip project neurites through hydrogel-filled microneedle arrays	[17]
Epicortical Surface-placed biohybrid interfaces	Cell-seeded on the cortex	Optical & electrical recording	–		Primary cortical neurons, hiPSC-derived neurons	Engineered neurons seeded on cortical surface for neurite-penetration based access of deep brain layers	[24]
	Cell outgrowth through perforated electrode	Electrical recording and stimulation	19 channels	115 kΩ @ 1 kHz	–	Proof-of-concept demonstration of the cell-through device without in vitro/in vivo demonstration	[154]
	Patterned cells on cranial window	Optical stimulation	118,000 microwells-	–	Primary cortical neuron	One neuron per well 2D microwell-integrated cranial window	[19]
3D culture integrated system	Spheroid axon guidance hydrogel tube	Electrical stimulation & calcium imaging	16 wells	8 kΩ @ 1 kHz	RGC, cortical spheroid	Electrically isolated individual neural spheroids with axonal projection to deep brain structure for synaptic relay	[20]
	Cortical organoid graft interface	Electrical recording	16 channels	1.4 MΩ @ 1 kHz	hiPSC-derived organoid	Monitoring the implanted organoid forming bidirectional synaptic connections with the host brain with simultaneous transparent microelectrodes and two-photon imaging	[21]

## Challenges and limitations

Despite substantial progress made in the biohybrid neural interface field, several challenges must be addressed to translate prototypes into durable implants. Key priorities include achieving target-specific axonal outgrowth and controlled synaptogenesis through site-selective culture and guided connectivity, maintaining long-term stability with high-fidelity electrode–neurite coupling and reliable high-SNR recordings, and developing decoding frameworks that remain robust to post-implantation circuit remodeling. In parallel, sustaining viability and immune acceptance in the host brain will remain a critical constraint.

The active formation of host-interface networks carries the risk of creating unintended neural circuitry. This biological variability limits the reproducibility of the functionality of biohybrid interfaces. Ongoing synaptic plasticity during the maturation process can generate unpredictable neural connections over time, ultimately compromising the reliability of recorded signals. Ultimately, even if the recording performance and electrical stability of the implanted electrode and the viability of the implanted tissue are maintained through the biohybrid neural interface, it is difficult to stably obtain reliable signals due to the freely occurring neural circuit changes after implantation. To promote predictable connectivity in an effort to preserve signal fidelity over time, it will be increasingly important to enforce precise spatiotemporal control of neurite outgrowth and targeted synapse formation using engineered guidance strategies such as patterned ECM cues, microtopography, and neurotrophic gradients.

Achieving long-term, high-reliability biohybrid neural interfacing will require both high-fidelity electrode–neurite coupling and decoding frameworks that can track circuit remodeling over time. High-yield recordings with high SNR depend on site-selective culture and reliable, preferential neurite coupling to intended targets, supported by strategies such as aligning neurons and neurite outgrowth over or through electrode sites, cell-type–specific and target-directed seeding to promote intended connectivity, rigorous demixing of signals originating from implanted versus host circuits, and multimodal electrical–optical–chemical readouts to disambiguate sources and enable cross-validation. In parallel, signal decoding platforms must actively adapt to dynamic post-implantation circuit integration; AI-driven adaptive decoding approaches now being developed to compensate for micromotion and long-term drift in conventional in vivo interfaces provide a useful foundation [156–159]. Likewise, AI imaging–based cell-culture platforms used to assess differentiation state in vitro could be extended to monitor ongoing circuit maturation and remodeling in biohybrid neural interfaces, providing a technical basis for sustained, high-fidelity signal monitoring [160].

Biocompatibility and immune modulation remain critical: even with ultraflexible, brain-soft interfaces, long-term acceptance will likely depend on autologous or immune-evasive cell sources. Within this context, the added biological degrees of freedom in biohybrid neural interfaces introduce variability and new failure modes that must be understood and controlled, making clinical translation a longer-term objective than for conventional implantable neural interfaces and one that will require substantial foundational work. From a translational standpoint, a central unresolved question is whether adding a living cellular component net improves chronic electrode–tissue coupling once realistic sources of biological variability are considered—namely, immune recognition, maturation drift, viability constraints, and remodeling of both the graft and host tissue. Although living-cell integration is often motivated by the prospect of reducing chronic inflammation and fibrotic encapsulation, the immunological relationship among implanted cells, device materials, and host brain remains insufficiently characterized and is likely to be dynamic over time. Patient-derived iPSC neurons/glia, HLA-matched donor banks, and emerging immune-evasive products could, in principle, reduce immunogenetic mismatch and improve compatibility relative to mismatched allogeneic sources [161–163]. Related cell-therapy strategies are already being advanced clinically for tissue repair and neurodegenerative indications [164–169], underscoring both the motivation for patient-matched approaches and the stringent safety expectations for stem cell–derived products [170–173].

Overall, achieving reliable biohybrid neural interfaces implants will require co-optimizing living constructs with device materials and structures, where key challenges include controlling variability in neurite outgrowth and synaptogenesis, device–cell integration, adaptive decoding under circuit remodeling, and host immune interactions.

## Summary and future outlooks

The central message of this review is the convergence of *in vitro* MEAs and *in vivo* neural interfaces. MEA platforms, depending on the specific design, support massively parallel, network-level recording; precise, selective guidance of mono- and co-cultures; and intracellular recordings at scale, stable interfaces, and high-fidelity recording and stimulation. *In vivo* brain interface systems have made substantial advances, including expanded channel counts and mechanically compliant microscale electrodes, but micromotion, immune activation, and progressive long-term signal degradation continue to limit chronic performance. Living biomaterials bridge these domains, translating MEA-derived principles into implantable, adaptive biohybrid neural interfaces that integrate within the host. This convergence marks a shift from flexible and soft biotolerant devices toward genuinely biointegrative systems in which living cells help close the mechanical, electrical, and functional gap between device and tissue.

Looking ahead, the next leap will come from systematically exporting MEA innovations into *in vivo* biohybrid neural interfaces. Key opportunities include in situ neuronal guidance to steer axons and lock synapses at intended sites, tight coupling to CMOS back-ends for dense sensing/stimulation/closed-loop control, multicellular co-cultures of neurons, glia, and vascular/immune support cells to sustain metabolism and longevity, and nanostructure-integrated interfaces for intracellular-like access while preserving chronic stability.
